# The Effect of Exercise During Pregnancy on Maternal and Offspring Vascular Outcomes: a Pilot Study

**DOI:** 10.1007/s43032-020-00302-7

**Published:** 2020-11-30

**Authors:** Áine Brislane, Helen Jones, Sophie M. Holder, David A. Low, Nicola D. Hopkins

**Affiliations:** 1 Research Institute of Sport and Exercise Sciences, Liverpool John Moores University, Liverpool, L3 3AF UK; 2 School of Sport, York St. John University, Lord Mayor’s Walk, York, YO31 7EX UK

**Keywords:** Pregnancy, Exercise, Cerebrovascular function, Endothelial function, Offspring

## Abstract

The aim of this pilot study is to obtain estimates for the change in maternal cerebrovascular (primary) and offspring vascular structure (secondary) during healthy pregnancy that includes structured exercise. Eighteen pregnant women self-assigned to a moderate-intensity aerobic exercise intervention or a control group. Maternal cerebral blood flow (CBF) at the middle cerebral artery, cerebro- and peripheral-vascular function was assessed at the end of each trimester. Offspring carotid artery intima-media thickness (IMT) was measured within 12 weeks of birth. For exploratory purposes, we performed statistical analysis to provide estimates of the change for primary and secondary outcome variables. Maternal CBF reduced (− 8 cm s^−1^ [− 14 to − 2]) with evidence of change to cerebral autoregulation (normalised gain: 0.12 %cm s^−1^% mmHg^−1^mmHg/% [− 0.18 to 0.40]) during pregnancy. Offspring carotid IMT was smaller in the exercise group (− 0.04 mm [− 0.12–0.03]) compared with controls. Based upon this data, a sample size of 33 and 57 in each group is required for low-frequency normalised gain and offspring IMT, respectively. This would provide 90% power to detect statistically significant (*P* < 0.05) between group differences in a randomised controlled trial. CBF is reduced in pregnancy, possibly due to reduced vascular resistance and altered maternal cerebral autoregulation. Maternal exercise had negligible effects on cerebrovascular adaptation to pregnancy, but we observed lower offspring carotid artery wall thickness following maternal exercise. Our directional findings and sample size estimations should be explored in a fully powered randomised control trial.

Clinical trial registration: The trial was registered on March 14th at https://register.clinicaltrials.gov (NCT03079258). Participant enrolment began on 3rd April 2016.

## Introduction

Pregnancy is associated with significant cardiovascular (CV) adaptations including increased cardiac output, blood volume and vasodilation alongside decreased mean arterial pressure [1]. Increased vasodilation is critical for adequate delivery of oxygen and nutrients to the foetus and is achieved in part, via an increased production of vasoactive substances such as nitric oxide (NO) at the vascular endothelium [2]. Underlying undetected endothelial injury may impede essential vascular adaptation during pregnancy and plausibly underpin the pathophysiology of cerebrovascular [3] and peripheral vascular pregnancy-related complications [4]. For example, cerebrovascular risk is four-to-six times higher in women with pregnancy-related hypertensive disorders [5] and this is expected to rise due to maternal obesity and older age in pregnancy [6]. Improving our understanding of pregnancy-related cerebrovascular adaptation is therefore warranted.

Maternal cerebral blood flow (CBF) declines during pregnancy due to reduced blood pressure (BP) and vascular resistance [7]. The regulation of CBF in response to changes in carbon dioxide (CO_2_) and BP, termed cerebral autoregulation (CA), appears altered over the course of a single healthy pregnancy [1, 8]. The upper and lower limits of CA are extended beyond the non-pregnant range (60–160 mmHg) in an animal model which is likely a protective mechanism against risk of pregnancy-related ischemic brain injury in response to changes in BP [8]. An understanding of the pregnancy-related changes in CA at each trimester of healthy human pregnancy is required to advance current knowledge of the cerebrovascular risk that accompanies complicated pregnancies. Similarly, the time course of several structural and functional conduit artery vascular adaptations, including brachial artery flow-mediated dilation (FMD) and artery size (diameter), thickness (carotid intima media thickness (IMT)) and stiffness, have not been collectively described longitudinally in a single cohort of pregnant women [9–13]. Characterising the integrity of the systemic vasculature during a healthy pregnancy would allow for future comparison of the pathophysiological changes that result in gestational diabetes and pre-eclampsia onset, both of which have long-term consequences for maternal cerebro- and peripheral-vascular disease risk [2].

Exercise is a modifiable lifestyle behaviour known to positively influence cerebro- [14] and peripheral vascular function in healthy adults [15]. Similarly, evidence suggests that exercise during pregnancy improves brachial FMD during the third trimester [16]. Importantly, the CV benefits of gestational exercise may also translate to the offspring. Maternal exercise positively impacts progeny heart rate [17] and femoral artery endothelial cell function in swine offspring [18]. Maternal exercise has also shown to induce long-term vascular programming into adulthood [19]. These data suggest that exercise during pregnancy may program vascular adaptations in offspring as well as the mother; however, this has yet to be investigated in a human cohort. Therefore, the aims of this pilot study are to obtain estimates for the change in maternal cerebrovascular (primary) and offspring vascular function (secondary) during healthy pregnancy that includes structured exercise.

## Materials and Methods

### Participant Characteristics

**Fig. 1:**
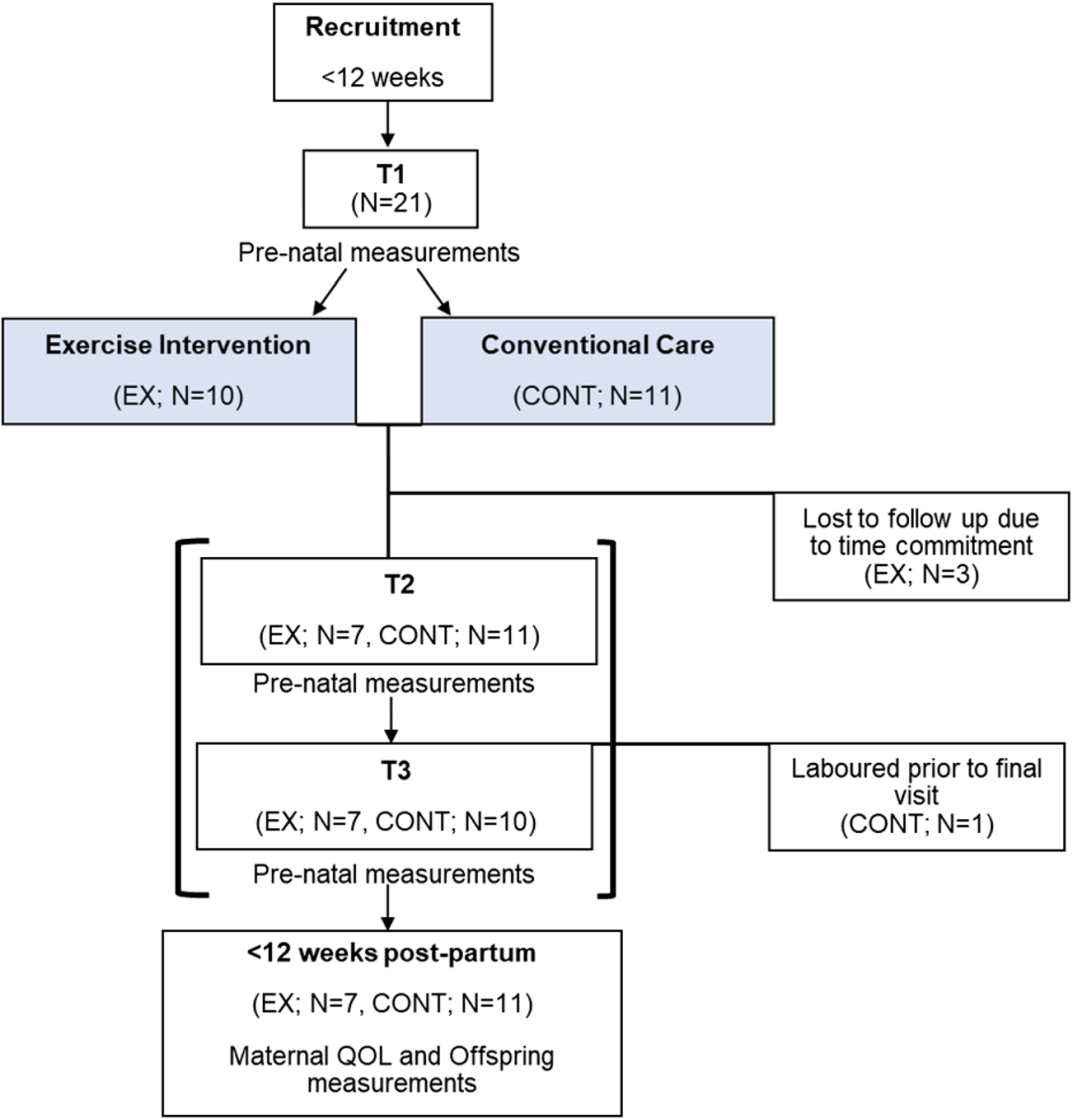
Schematic of the experimental procedure used. Participants were recruited within 12 weeks of pregnancy for vascular assessments. Participants chose to join an exercise intervention or continue with conventional care and made two subsequent visits at the end of T2 and T3 for vascular measurements. Within 12 weeks post-partum, offspring vascular measurements were obtained

Twenty-one singleton pregnant participants were recruited at local pre-natal clinics between February 2017 and May 2018 (Fig. 1). Women were eligible if they were in the first trimester of pregnancy, non-smokers for at least 6 months, had no history of CV disease, gestaional diabetes mellitus (GDM) or pre-eclampsia, were not on any form of medication, participated in structured exercise less than twice/week and had a body mass index (BMI) < 35 kg m^−2^. The inclusion of women undertaking less than two exercise sessions per week prior to their pregnancy was used as an indicator of habitual exercise because the intervention implemented was in line with previously published guidelines by the Royal College of Obstetrics and Gynaecology for previously inactive pregnant women [20]. Women were excluded from the study if they developed any complication such as pre-eclampsia, gestational diabetes or had incidence of intrauterine growth restriction. All participants provided written consent before taking part in the experimental procedure. Offspring characteristics including sex, gestational age at birth and birth weight, alongside delivery method including vaginal, C-section, ventouse and forceps were collected from midwives using patient notes. This research study was ethically approved by the National Health Service Liverpool Central Research Ethics Committee (Reference: 17/NW/0056), adhered to the Declaration of Helsinki and was registered as a clinical trial at ClinicalTrials.gov NCT03079258.

### Experimental Procedure

Participants were required to visit the temperature-controlled (20–22 °C) laboratory on three occasions having abstained from exercise for 24 h and alcohol for 12 h as well as any food/caffeine/stimulants 6 h prior to the experiment. Each visit was completed at the same time of day within-participants and corresponded closely with the end of trimester 1 (T1; 12–14 weeks), trimester 2 (T2; 24–26 weeks) and trimester 3 (T3; 36–38 weeks). Participants completed a battery of CV and cerebrovascular assessments in the following order: neurovascular coupling (NVC), IMT, FMD, pulse wave velocity (PWV), cerebrovascular reactivity (CVR) and CA. The Astrand submaximal cycling test was performed to estimate cardiorespiratory fitness (estimated VO2_peak_). This test was chosen due to associated low level of exertion and to minimise any injury risk that might compromise exercise participation during the study. Following each visit, participants recorded 7 days of objective physical activity (PA) and sedentary behaviour monitoring. At the initial visit (T1), participants chose to take part in a partially supervised exercise (EX) intervention or to participate as a control (CONT). Those in the control group received conventional care that involved routine clinical visits with no interaction with the research team or the exercise group. Routine clinical advice given to women by midwives at the hospital was to refrain from starting exercise in pregnancy if previously inactive, and as such, no women in the CONT group were routinely exercising. Within 12 weeks of birth, offspring artery structure was measured and hospital records were used to acquire offspring characteristics and delivery information via the midwifery team.

### Pre-natal Measurements

#### Cerebrovascular Measurements

##### MCA and PCA Insonation

Participants lay in a semi-recumbent position and were fitted with a 2-MHz pulsed transcranial Doppler (TCD) ultrasound system (DWL, Compumedics, Germany) comprising a headband which supports an ultrasound probe on each side of the head. Ultrasound gel was applied to the temporal window (just above the zygomatic arch) and to the probes allowing an optimal signal to be obtained. On either side of the cerebral headband, the posterior cerebral artery (PCA) and middle cerebral artery (MCA) were insonated, respectively, as described elsewhere [21]. Care was taken to stabilise the probes ensuring a stable angle of insonation in line with best practice guidelines [22]. Additionally, participants were instrumented with a mouthpiece, nose clip, a bacteriological filter and a three-way valve to allow for PETCO_2_ monitoring. Simultaneously, arterial blood pressure was monitored non-invasively using a Finometer photoplethysmography (Finometer pro, Finapres Medical Systems, Netherlands) which was carefully fitted on either the right hand’s middle or index finger as per manufacturer’s recommendations. Real-time MCAv and BP were recorded online and displayed in LabChart Pro version 7 (ADInstruments, Australia). The weighted mean MCAv and PCAv were calculated from the peak envelope of the velocity trace (1/3 systolic + 2/3 diastolic), which accounts for the relative time spent in each phase of the cardiac cycle [23]. Data were expressed as cerebrovascular conductance (CVC), which was calculated as MCAv divided by mean arterial pressure (1/3SBP + 2/3DBP).

##### Neurovascular Coupling

A 120-s baseline was recorded with participant’s eyes open, followed by 120 s of eyes closed to act as a familiarization phase. Participants were then asked to open and close their eyes in 30-s intervals for a period of 5 min as prompted by the researcher. During ‘eyes open’, participants were instructed to fixate on a red dot in the centre of a moving black and white checkered image. The video was displayed on an iPad (Apple) and held ~ 7 cm away from their eyes to reduce the effects of external light. The same side for MCA and PCA were insonated for repeated measurements to ensure consistency. The visual stimulation used provides a strong mechanistic model of neurovascular coupling by increasing neural activation of the occipital lobe [24].

Data was anaylsed following recommended guidelines using an automated software [24]. First analysis of NVC was performed by visually inspecting recordings on LabChart for noise in the signals and artefacts. Interpolation was used to replace any short segments and any larger segments were excluded. Data were extracted from LabChart on a beat-to-beat basis (peak, minimum and mean MCAv & PCAv along with BP, PETCO_2_ and HR). Only the 5 cycles of 30-s eyes-open-eyes-closed were extracted for analysis. Data was run through the MATLAB script where the software combines all the cycles into one average contour for each physiological recording of each eyes-open-eyes-closed cycle. Time to peak response, absolute and percentage change in the PCAv were used to quantify the NVC response.

##### Cerebrovascular Reactivity

Bilateral MCAv was employed to assess CVR to perturbations in PaCO_2_ using a hypercapnic rebreathing protocol described elsewhere [25]. In brief, participants were instrumented again with a mouthpiece, nose clip, a bacteriological filter and a three-way valve to allow switching of airflow between room air and a pre-filled Douglas bag containing a hypercapnic gas mixture of 5% CO_2_, 21% O_2_ balanced with nitrogen. Breath-by-breath CO_2_ was sampled using a calibrated gas analyzer (Ml206, ADInstruments) and the pressure of end-tidal carbon dioxide (PETCO_2_) was recorded online (LabChart) and corrected for the daily barometric pressure. A 1-min baseline recording was followed by a period of voluntary hyperventilation until a reduction in PETCO_2_ to < 20 Torr. Once achieved, participants inhaled the 5% CO_2_ mixture from the Douglas bag and instructed to return their respiratory rate to normal for 3 min. Data was exported from LabChart Pro (ADInstruments Australia) and analysed as previously described [26]. CVR was quantified by linear regression (*R*^2^ value). Relative MCAv was calculated as the difference between baseline and 5% CO_2_ MCAv divided by baseline MCAv (([5% CO_2_ MCAv-baseline MCAv] / baseline MCAv) × 100%)***.*** Simultaneously during the CVR test, CCA diameter was acquired at least 2 cm below the point of bifurcation using high-resolution ultrasound. Images were acquired in accordance with methodological guidelines [27]. Data were used to determine the response of the CCA to elevations in PETCO_2_ by averaging 30 s of baseline diameter and comparing that to the diameter during the last 30 s of 5% CO_2_ breathing.

##### Cerebrovascular Autoregulation

Changes in BP and MCAv were assessed using a squat to stand procedure in order to induce transient changes in arterial BP. Participants performed squat-stand maneuvers at 0.10 Hz (5 s squat and 5 s stand) whilst breathing normal atmospheric air for a duration of 6 min with PETCO_2_ monitored throughout. MCAv, PETCO_2_ and MAP were extracted from LabChart every 0.1 s across the 6-min period. The relationship between changes in MCAv and arterial BP was assessed via the transfer function analysis (TFA) in accordance with standardised guidelines [28]. Transfer function analysis was performed using MATLAB (MathWorks-Inc., Natick, MA) in order to calculate associated power (gain) and timing (phases) over three different frequencies; very low (0.02–0.07 Hz), low (0.07–0.20 Hz) and high (0.20–0.50 Hz) [28]. TFA also produces an estimated reliability of the relationship between the two signals (coherence) [29]. Data sets with a coherence value of < 0.4 Hz were excluded from data analysis. High frequency and very low frequency range data were excluded from analysis based on the frequency of the squat-stands used.

### Vascular Structure and Function

#### Carotid and Femoral Intima-Media Thickness

Following 20 min of rest in a semi-recumbent position, the left common carotid artery (CCA) was imaged using high-resolution B-mode ultrasound (Terason u-smart 3300, Teratech, Burlington, MA, USA) from three angles (approximately 45°, 90° and 135°), 5 mm proximal to the artery bulb [30]. Images were optimised to ensure clear contrast between the artery walls and lumen with a distinct IMT visualised on the far wall defined as the distance between two echogenic lines represented by the lumen-intima interface and media adventitia interface of the artery wall. The IMT was also acquired at the left femoral artery 3 to 5 cm distal to the bifurcation using the same criteria as for CCA. Each image was recorded by the same sonographer for 30–40 s.

Recordings were analysed offline using the edge detection software Carotid Studio v4.3.1 (Cardiovascular Suite, Quipu srl, Pisa, Italy) with a frame rate of 25 frames per second as described elsewhere [31]. Continuous calculations by the software produced an average IMT and artery diameter recorded and was repeated for all three angles and an average of the three angles was calculated. This method has been shown to be valid and reproducible. The extracted data were also used to calculate wall-to-lumen ratio (IMT/lumen) at each arterial site which corrects for differences in baseline diameter.

#### Arterial Stiffness

Carotid-femoral PWV was assessed using a semi-automated device and software (SphygmoCor, AtCor Medical, Sydney, Australia) in the semi-recumbent position as previously reported in pregnant populations [32]. Firstly, three brachial artery BP measurements were taken in succession (Dinamap V100, GE Medical Systems, Germany), with an average systolic (SBP) and diastolic (DBP) calculated and entered into the software. A single applanation tonometer probe was used to capture a proximal (carotid artery) and distal (femoral artery) pulse, recorded over 10 cardiac cycles. The QRS complex was measured simultaneously using electrocardiography (ECG). The time between the R wave of the ECG trace and the foot of the proximal waveform is subtracted from the time between the R wave and the foot of the distal waveform to obtain the pulse transit time. To determine the distance used for PWV, the distance from the proximal measurement site to the suprasternal notch was subtracted from the distance between the distal measurement site and the suprasternal notch using callipers. PWV was automatically calculated by dividing the distance between the two arterial recording sites by transit time to provide an index of arterial stiffness. PWV measurements were made in triplicate and an average was calculated and used in data analysis.

#### Brachial and Femoral Flow-Mediated Dilation

Left brachial and left femoral artery diameters were assessed simultaneously via high-resolution 2D duplex ultrasound (Terason u-smart 3300, Teratech) with a 10–12 MHz linear array probe. B-mode images were obtained and optimised, and the probe was held in the same position for the duration of the test. After 1 min of baseline measurement, occlusion cuffs, connected to a rapid inflator (Hokanson, Bellevue, WA), placed around the left thigh, proximal to the patella, and the left forearm, distal to the humeral epicondyle, were inflated to a supra-systolic pressure of 220 mmHg for 5 min. Arterial images were recorded for a further 3-min post cuff deflation in accordance with best practice guidelines [33].

Brachial FMD (bFMD) and femoral FMD (fFMD) data were analysed as described elsewhere [34] using a validated custom edge detection and wall tracking software (Dicom Encoder) [35]. FMD data was analysed with covariate control for baseline artery diameter (adjusted FMD) allowing FMD to be scaled for changes in artery diameter [36].

#### Anthropometry and Body Composition

Stature and body mass were recorded to the nearest 0.1 cm using a stadiometer and digital scales, respectively. Body mass index (BMI) was calculated as body mass in kilograms divided by stature in metres squared (kg m^−2^). Skinfold thickness measurements were performed at the biceps, triceps and subscapular landmarks as described elsewhere [37] and in line with the International Standards for Anthropometric Assessment Manual to calculate body fat percentage (BF%) [38].

#### Physical Activity

Physical activity was monitored using a tri-axial accelerometer (Actigraph wGT3x-BT). Participants wore the accelerometer on their right hip for 7 consecutive days at the end of T1, T2 and T3. Participants removed the device for sleeping, contact sports and water-based activities and recorded the times the device was worn on a diary sheet provided. Non-wear time was defined as 90 consecutive minutes of zero counts min^−1^ [39]. Inclusion criteria for analysis were ≥ 10 h of wear time per day, for a minimum of 4 days, including one weekend day [40]. The Actilife software, version 6.2 (ActiGraph, Pensacola, Florida), was used to download the data. Raw acceleration data was converted to 60-s epoch activity count data (counts min^−1^). PA intensity was determined using the following cut points: light (≤ 2689 counts min^−1^), moderate (≤ 6166 counts min^-1^) and vigorous (> 6167 counts min^−1^) [41]. Activity data were exported and handled in Excel (Microsoft) and total time (minutes) spent daily in light, moderate and vigorous activity was calculated.

#### Sedentary Behaviour

Sedentary time was objectively measured using an activPAL activity monitor (activPAL micro, PAL Technologies Ltd., Glasgow, UK) worn continuously for 7 days on the middle anterior of the right thigh. Monitors were enclosed in a rubber sleeve and attached by the researcher to the skin using a waterproof transparent seal (Tegaderm Roll, 3M). The monitor quantified the time spent sitting, lying, standing and walking per day. A sedentary bout was defined as no activity registered for at least 60 s [42]. The raw data was downloaded from the monitor using the activPAL proprietary software (version 7.2, 32) from which data were exported to Excel (Microsoft, UK). Seconds of sedentary time during waking hours were totalled and converted to minutes/day.

#### Cardiorespiratory Fitness Test

Participants performed a submaximal cycling test to estimate VO_2peak_ as previously described [43]. In brief, participants cycled at a self-selected resistance and pace for 2-min to be familiarised to the cycle ergometer. HR was monitored continuously throughout the test using short-range telemetry (Polar H7, Kempele, Finland) and noted at the end of each minute alongside RPE. The test began with participants cycling with a load of 60 W (W) for a period of 6 min. Participant’s VO_2peak_ was estimated using the Astrand-Rhyming nomogram based on mean HR (corrected for age) in the final minute of the test and the mechanical load at that point. Estimated absolute and relative VO_2peak_ was calculated and reported.

### Exercise Intervention

Participants began the exercise intervention after completing PA and sedentary behaviour monitoring in T1 (12–14 weeks) and continued until delivery at the end of T3. The intervention commenced with 3 × 15-min continuous exercise sessions at the beginning of T2, 3 × 30 min by the end of T2 and 4 × 30 in T3 in line with previously published pregnancy exercise guidelines by the Royal College of Obstetrics and Gynaecology (RCOG) [20]. The authors acknowledge that these guidelines have been updated; however, this study had commenced prior to the release of the updated guidelines. The intervention comprised aerobic exercise performed on ergometer equipment at 60–70% estimated maximum heart rate (MHR). To account for pregnancy-related changes to heart rate, the Borg rate of perceived exertion scale [44] was also used. Participants were asked to exercise to a rating of 12–14 equating to some breathlessness although capacity to maintain a conversation in line with guidelines at the time [20]. One session per week was supervised by the researcher at the Liverpool John Moores University gym and the remaining sessions were completed at a Liverpool City Council gym for which memberships were provided. Participants wore a Polar Bluetooth Heart Rate Monitor (H10 Kempele, Finland) during all sessions and HR was recorded via a personal Polar Beat account which was remotely accessible to the researcher. Compliance was verified by totalling the number of sessions attended by each participant divided by the minimum number of sessions achievable in that time and is reported as a percentage. Adherence to the exercise was checked by verifying the heart rate recorded for each session by the Polar monitor. A session was adhered to if HR was equal to or greater than 60% estimated MHR.

### Post-natal Measurements

#### Offspring Carotid Intima-Media Thickness

Offspring carotid IMT was measured within 12 weeks of delivery. The common carotid artery was imaged using high-resolution B-mode ultrasound (Terason u-smart 3300, Teratech, Burlington, MA, USA) 5 mm proximal to the artery bulb [30]. Offspring were held supine or semi-supine by a parent with the neck slightly extended and where possible, facing the contralateral side to allow for optimal longitudinal imaging of the far-wall intima media interface at one angle only due to the small neck space available to scan. Images were recorded for 30–40 s and optimised to ensure clear contrast between the artery walls and lumen with a distinct IMT visualised on the far wall as described above. All offspring underwent a carotid artery scan; however, due to quality issues, one scan (EX group) was omitted from the analysis.

From the cIMT recording, 6–8 s of clear IMT and artery walls was selected for analysis using an edge detection software (Motion JPEG Encoder V3, National Instruments, USA) as described elsewhere [45]. This software allows frame by frame viewing to ensure tracking of the artery wall and IMT which was necessary given the movement from offspring during scanning. Each frame was checked by the same sonographer, to ensure the diameter and IMT had been registered correctly, and any tracking mistakes made by the software were deleted. The extracted data were used to calculate IMT/lumen to account for differences in baseline diameter. This method is reliable and operator-independent, demonstrating high levels of precision and accuracy for estimating conduit artery diameter and wall thickness [46].

### Statistical Analysis

Given that this is a pilot study to obtain estimates of primary and secondary outcome variables, no a priori sample size was calculated. The primary outcome in the study is low-frequency normalised gain, a marker of dynamic cerebral autoregulation and the primary comparison is between T2 and T3. These time points were chosen to capture a large proportion of the vascular adaptation in pregnancy (T2) [47] and secondly, because of the association between altered CA in late pregnancy (T3) [48]. Using the data collected during the study, we calculated post hoc power, and also the required sample size for a future, fully powered randomised control trial for both primary and secondary outcome variables (G*Power 3.1.5).

For exploratory purposes, we performed statistical analysis to provide an estimate of the changes in all outcome variables (Statistical Package for Social Sciences). Baseline (T1) participant characteristics were compared using an independent *t* test. Cerebrovascular parameters, FMD, IMT, cardiorespiratory fitness and PA were analysed using linear mixed modelling. Significant interactions and main effects were followed up using LSD pairwise comparisons. An independent sample *t* test was used to identify any differences between EX and CONT group offspring IMT. Data are presented in the text as mean [95% confidence interval] unless otherwise stated, with exact *p* values. *P* values are presented but not interpreted.

## Results

### Participant Characteristics

On entry to the study, groups were similar in age, BMI, BF, fitness and blood pressure (Table 1). Of the 21 women enrolled in the study, 3 withdrew (2 from the EX group) due to time commitment and 1 (CONT) laboured prior to the final measurement. None of the participants developed any pregnancy-related complications during this study. Body mass and BMI increased by 10.7 kg [5, 17] and 4 kg m^−2^ [2, 6], respectively, during pregnancy (Table 1) but similarly with each intervention. There were negligible changes in SBP, DBP, MAP and fitness change during pregnancy.

**Table 1:** Descriptive characteristics of participants in the exercise and control group at the end of trimester 1, 2, and 3

	Exercise group (*N* = 7)			Control group (*N* = 11)			Two-way ANOVA		
Trimester	T1	T2	T3	T1	T2	T3	Time	Intervention	Time × intervention
Age (years)	33 ± 4	33 ± 4	33 ± 2	33 ± 3	33 ± 3	34 ± 2	0.96	0.97	0.99
Body mass (kg)	62.0 ± 8.4	69.5 ± 8.6	73.3 ± 9.7	66.6 ± 7.7	73.0 ± 7.3	76.5 ± 6.6	0.01	0.24	0.99
BMI (kg m^−2^)	23 ± 3	26 ± 3	27 ± 4	24 ± 3.	26 ± 3	28 ± 3	0.01	0.87	0.96
Body fat (%)	22.3 ± 2.2	24.6 ± 3.7	25.4 ± 4.2	23.5 ± 3.7	25.1 ± 3.7	25.9 ± 4.2	0.11	0.95	0.97
SBP (mmHg)	99 ± 6	102 ± 7	104 ± 6	103 ± 12	105 ± 13	104 ± 7	0.68	0.36	0.69
DBP (mmHg)	60 ± 8	61 ± 6	66 ± 6	60 ± 6	60 ± 6	62 ± 7	0.17	0.38	0.64
Absolute V̇O_2peak_ (L min^−1^)	2.3 ± 0.6	2.5 ± 0.5	2.5 ± 0.4	2.5 ± 0.7	2.5 ± 0.7	2.5 ± 0.7	0.91	0.95	0.75
Estimated V̇O_2peak_ (mL kg min^−1^)	35.3 ± 10.2	34.0 ± 8.9	32.7 ± 7.5	36.7 ± 12.3	33.3 ± 11.4	30.9 ± 10.4	0.38	0.77	0.81

Values are mean ± SD. Italics represent significant main effect (*p* < 0.05)

*T1* trimester 1, *T2* trimester 2, *T3* trimester 3, *BMI* body mass index, *SBP* systolic blood pressure, *DBP* diastolic blood pressure, *V̇O*_*2*_ oxygen consumption

### Exercise Intervention

Overall compliance to the EX intervention was 78% (T2, 84% and T3, 68%). The EX group engaged in less sedentary time (456 min day^−1^ [411, 501] compared with the CONT group (523 min day^−1^ [485, 562]; *p* = 0.03), but there were no changes during pregnancy or with intervention. Light, moderate, vigorous and total PA or accelerometer wear time were similar during pregnancy across both interventions (*p* > 0.05; Table 2).

**Table 2:** Physical activity and sedentary behaviour for participants in the exercise and control group and the end of trimester 1, 2, and 3

	Exercise group (*N* = 7)			Control group (*N* = 11)			Two-way ANOVA		
Trimester	T1	T2	T3	T1	T2	T3	Time	Intervention	Time ×intervention
Sedentary time (min day^−1^)	464 ± 101	445 ± 85	458 ± 132	534 ± 110	553 ± 72	484 ± 103	0.70	0.03	0.49
Step count	5626 ± 1304	6051 ± 2315	6141 ± 1965	6072 ± 2254	5017 ± 1513	5136 ± 1822	0.89	0.33	0.45
Light PA (min day^−1^)	335 ± 79	346 ± 60	315 ± 97	322 ± 88	299 ± 76	301 ± 57	0.75	0.27	0.74
LPA % Wear time	39 ± 7	42 ± 5	38 ± 8	40 ± 12	36 ± 9	39 ± 8	-	-	-
Moderate PA (min day^−1^)	29 ± 16	27 ± 19	30 ± 19	30 ± 9	25 ± 13	24 ± 24	0.71	0.78	0.81
MPA % Wear time	3 ± 2	3 ± 2	4 ± 2	4 ± 2	3 ± 2	3 ± 3	-	-	-
Vigorous (min day^−1^)	1 ± 2	1 ± 2	1 ± 2	1 ± 2	0 ± 1	1 ± 1	0.76	0.58	0.69
VPA % Wear time	0 ± 0	0 ± 0	0 ± 0	0 ± 0	0 ± 0	0 ± 0	-	-	-
Total PA (min day^−1^)	364 ± 92	373 ± 75	345 ± 106	355 ± 100	323 ± 83	326 ± 66	0.75	0.30	0.77
Avg. daily wear time (min day^−1^)	847 ± 91	824 ± 78	814 ± 92	822 ± 89	848 ± 118	771 ± 76	0.30	0.59	0.59

Values are mean ± SD. Italics represent significant main effect (*P* < 0.05)

*T1* trimester 1, *T2* trimester 2, *T3* trimester 3, *PA* physical activity, *LPA* light physical activity, *MPA* moderate physical activity, *VPA* vigorous physical activity, *Avg* average

### Pre-Natal Measurements

#### Cerebrovascular Measurements

Low-frequency normalised gain changed by 0.12%cm s^−1^% mmHg^−1^mmHg/% [− 0.18, 0.40; main effect of time *p* = 0.08] during pregnancy. Our data provided 65% power to detect a between-group difference in low frequency normalised gain from T2 to T3. Using this data, a sample size of 33 in each group would provide 90% power to detect a statistically significant (*P* < 0.05) between intervention differences in low-frequency normalised gain in a future randomised controlled trial. Low-frequency phase increased by 13.9° [− 0.6, 28.5]; *p* = 0.06). These changes were similar for the exercise intervention (Table 6).

**Fig. 2:**
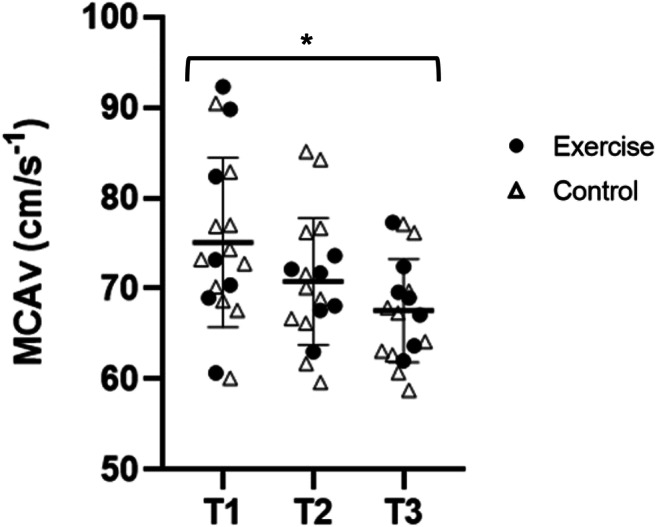
Maternal middle cerebral artery velocity during pregnancy. MCAv declined during pregnancy data for trimester 1, 2 and 3 which. *Main effect for time; *p* < 0.05

Resting MCAv reduced during pregnancy (− 8 cm s^−1^ [− 14, − 2]; *p* = 0.02; Fig. 2) and this was similar with exercise. PCAv during neurovascular coupling was higher in the EX group (43.7 cm s^−1^ [40.4, 47.0]) compared with the CONT group (39.2 cm s^−1^ [36.5, 41.9]; *p* = 0.04). There were negligible changes to cerebrovascular reactivity and associated parameters (basal PETCO_2_, linearity between MCAV and PETCO_2_, absolute CVR, relative CVR or MAP response during the CVR test) (Table 5).

#### Vascular Structure and Function

**Fig. 3:**
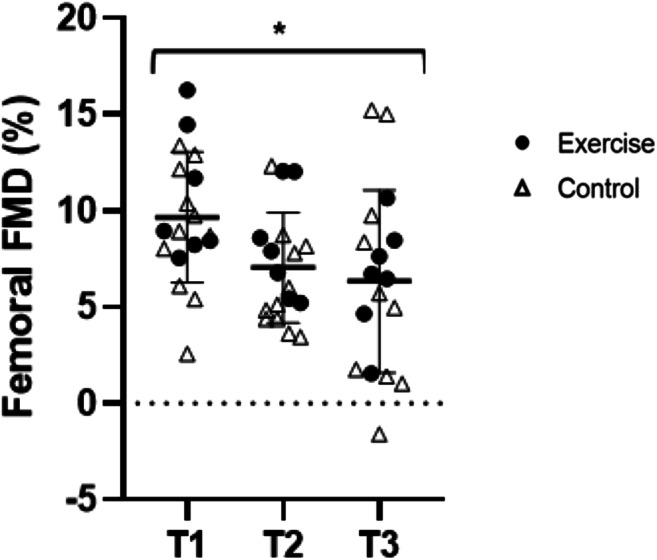
Femoral artery FMD during pregnancy. fFMD decreased progressively during pregnancy. *Main effect of time; *p* < 0.05

Brachial artery resting blood flow was higher in the EX group (1.60 ml s^−1^ [1.03, 2.17]) compared with the CONT group (0.80 ml s^−1^ [0.34, 1.27]; main effect of intervention *p* = 0.04). Brachial artery resting blood flow was not influenced by pregnancy (*p* = 0.66) and there was no time × intervention effect present (*p* = 0.76). Brachial artery diameter increased during pregnancy (0.03 cm [0.01, 0.06]; *p* = 0.03) and this was similar with exercise. Brachial FMD, allometrically scaled bFMD, TTP and SR_AUC_ were unchanged with pregnancy and exercise. Femoral artery FMD decreased in pregnancy (3.5% [− 6.5, − 0.5]; *p* = 0.03; Fig. 3) and was similar with interventions. Femoral artery resting blood flow, FMD, allometrically scaled fFMD, femoral artery diameter, TTP or SR_AUC_ were unchanged with pregnancy and exercise (*p* > 0.05; Table 3).

**Table 3:** Brachial and femoral artery function for exercise and control group at the end of trimester 1, 2 and 3

	Exercise group (*N* = 7)			Control group (*N* = 11)			Two-way ANOVA		
Trimester	T1	T2	T3	T1	T2	T3	Time	Intervention	Time × intervention
Brachial artery									
Baseline flow (ml s^−1^)	1.6 ± 2.8	1.3 ± 1.3	1.9 ± 1.3	0.7 ± 0.8	0.8 ± 0.9	0.9 ± 0.9	0.66	0.04	0.76
Artery diameter (cm)	0.31 ± 0.04	0.32 ± 0.03	0.35 ± 0.04	0.30 ± 0.03	0.32 ± 0.04	0.32 ± 0.03	0.03	0.24	0.69
Peak artery diameter (cm)	0.33 ± 0.04	0.35 ± 0.03	0.39 ± 0.04	0.32 ± 0.03	0.35 ± 0.04	0.35 ± 0.04	0.02	0.16	0.29
Time to peak (s)	44 ± 11	37 ± 13	63 ± 21	66 ± 27	49 ± 23	49 ± 10	0.18	0.39	0.10
SR_AUC_ (× 10^3^)	17.2 ± 10.0	15.8 ± 8.2	23.0 ± 9.3	20.9 ± 6.8	19.3 ± 10.9	22.9 ± 6.0	0.17	0.33	0.74
FMD (%)	7.1 ± 3.4	7.6 ± 4.0	10.8 ± 5.0	8.1 ± 2.9	7.6 ± 2.3	6.8 ± 3.8	0.60	0.30	0.16
Adjusted FMD (%)	7.1 ± 2.1	7.5 ± 2.2	10.8 ± 2.4	7.9 ± 1.9	7.6 ± 1.8	6.6 ± 2.0	0.62	0.27	0.14
Femoral artery									
Baseline flow (ml s^−1^)	0.9 ± 0.4	0.6 ± 0.3	0.7 ± 0.2	1.9 ± 2.1	0.8 ± 0.5	0.7 ± 0.6	0.23	0.14	0.40
Artery diameter (cm)	0.52 ± 0.04	0.54 ± 0.05	0.54 ± 0.05	0.51 ± 0.04	0.54 ± 0.05	0.54 ± 0.05	0.19	0.91	0.92
Peak artery diameter (cm)	0.58 ± 0.04	0.59 ± 0.05	0.57 ± 0.05	0.56 ± 0.04	0.57 ± 0.04	0.57 ± 0.06	0.72	0.51	0.83
Time to Peak (s)	64 ± 19	68 ± 44	105 ± 41	65 ± 34	55 ± 26	62 ± 27	0.15	0.06	0.17
SR_AUC_ (× 10^3^)	18.0 ± 5.0	13.7 ± 3.8	15.1 ± 5.3	23.3 ± 12.8	16.2 ± 7.2	16.5 ± 4.9	0.15	0.15	0.75
FMD (%)	10.8 ± 3.4	8.3 ± 2.9	6.6 ± 2.4	8.9 ± 3.3	6.3 ± 2.7	6.2 ± 5.9	0.03	0.19	0.84
Adjusted FMD (%)	11.2 ± 1.4	7.6 ± 1.5	6.5 ± 1.4	7.4 ± 1.8	6.1 ± 1.8	7.8 ± 1.8	0.02	0.18	0.88

Values are mean ± SD

*SR*_*AUC*_ shear rate area under the curve, *FMD* flow mediated dilation

^†^*p* < 0.05 for condition

Carotid artery diameter increased during pregnancy (0.33 mm [0.07, 0.58]; *p* = 0.04) and was similar with intervention. Carotid IMT did not change with pregnancy or exercise (*p* > 0.05; Table 4). Carotid IMT/lumen decreased during pregnancy (0.005 [− 0.01, 0.001]; *p* = 0.046) and was similar with intervention. Femoral artery diameter, fIMT and fIMT/lumen were unchanged with pregnancy and exercise (*p* > 0.05). PWV reduced during pregnancy (5.01 m s^−1^ [2.8, 7.2]; *p* = 0.09; Table 4). PWV was greater in the EX group (5.3 m s^−1^ [1.6, 8.9]) compared with the CONT group (4.7 m s^−1^ [3.0, 6.5]; main effect of intervention *p* = 0.04). There was no time × intervention effect for PWV (*p* = 0.68).

**Table 4:** Carotid and femoral artery structure and pulse wave velocity for exercise and control group at the end of trimester 1, 2 and 3

	Exercise group (*N* = 7)			Control group (*N* = 11)			Two-way ANOVA		
Trimester	T1	T2	T3	T1	T2	T3	Time	Intervention	Time ×intervention
Pulse wave velocity (m s^−1^)	5.36 ± 0.27	4.96 ± 0.71	5.51 ± 0.88	5.07 ± 0.57	4.49 ± 0.67	4.67 ± 1.29	0.09	0.04	0.68
Carotid artery									
Lumen artery diameter (mm)	6.90 ± 0.43	7.08 ± 0.56	7.18 ± 0.33	6.78 ± 0.32	7.10 ± 0.49	7.14 ± 0.39	0.05	0.72	0.90
IMT (mm)	0.47 ± 0.06	0.49 ± 0.06	0.47 ± 0.05	0.51 ± 0.04	0.49 ± 0.04	0.49 ± 0.04	0.54	0.73	0.29
IMT/lumen	0.07 ± 0.01	0.07 ± 0.01	0.06 ± 0.00	0.08 ± 0.01	0.07 ± 0.01	0.07 ± 0.01	0.046	0.19	0.21
Femoral artery									
IMT (mm)	0.40 ± 0.06	0.41 ± 0.04	0.41 ± 0.09	0.37 ± 0.07	0.39 ± 0.07	0.40 ± 0.05	0.64	0.41	0.97
IMT/lumen	0.07 ± 0.01	0.08 ± 0.01	0.07 ± 0.02	0.07 ± 0.01	0.07 ± 0.01	0.07 ± 0.01	0.96	0.55	0.84

Values are mean ± SD

*IMT* intima-media thickness

*p < 0.05 for time

### Post-natal Measurements

Gestational age born and birth weight were similar in the EX and CONT group. No birth defects or adverse events were reported during delivery. In the EX group, 3 babies were born by normal delivery, 2 by C-section (elective), 1 by ventouse and 1 by rotational forceps delivery. In the CONT group, 6 were born by normal delivery, 4 by C-section (3 electives, 1 emergency) and 1 induced to normal delivery. This equated to 26% C-section in the EX group and 36% in the CONT group.

**Fig. 4:**
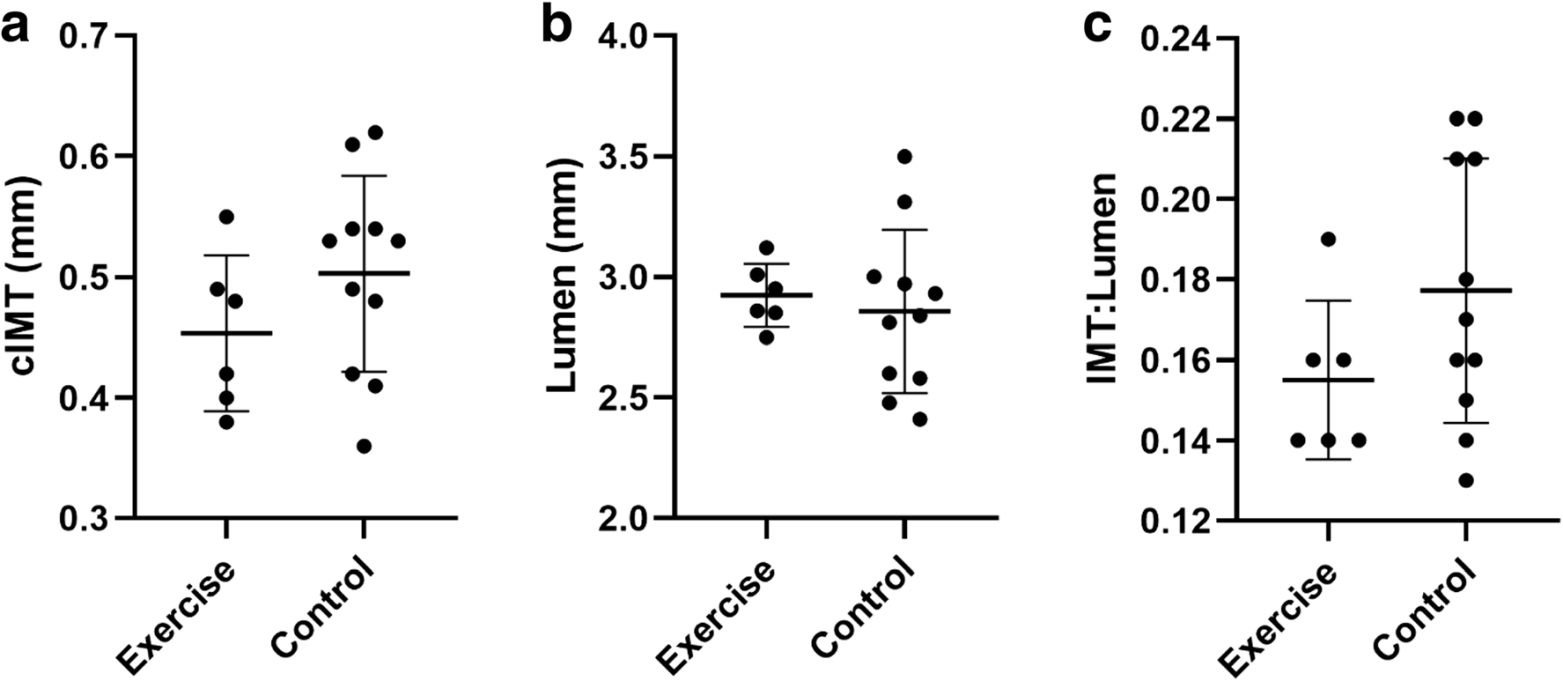
Offspring vascular outcomes data. Offspring cIMT (**a**) and IMT:lumen (**c**) are lower in offspring of exercising mothers compared with the control group, while the lumen diameter is larger (**b**) in exercisers compared with the control group although not significant

Offspring carotid IMT was smaller the exercise group compared with the control group (− 0.04 mm [− 0.12–0.03]. Our data provided 31% power to detect a between-intervention difference for offspring IMT. Using this data, a sample size of 57 in each group would provide 90% power to detect a statistically significant (*P* < 0.05) between-group difference in a randomised controlled trial. Carotid IMT/lumen and lumen diameter were similar between interventions (Fig. 4).

## Discussion

The aim of this pilot study was to obtain estimates for the change in maternal cerebrovascular (primary) and offspring vascular function (secondary) during healthy pregnancy that included structured exercise. We provide preliminary evidence that maternal cerebral autoregulation changes during pregnancy. This observation is alongside a decline in MCAv and reduced vascular resistance via increased carotid artery diameter. These changes were not impacted by exercise during pregnancy. We did observe evidence of lower offspring carotid artery diameter following maternal exercise. Our directional findings and sample size estimations should be explored in a fully powered randomised control trial.

We provide the preliminary evidence of changes in dynamic CA during each trimester of healthy human pregnancy. Normalised gain represents the dampening effect of CA on the magnitude of BP oscillations. Gain rises with increasing BP frequencies and when increased, is indicative of diminished dynamic CA efficiency [49]. In the context of our findings, this implies that BP oscillations increase during pregnancy, resulting in a greater magnitude between BP and MCAv, such that CA efficiency is reduced. The second CA parameter, phase, describes the synchronicity of oscillations of BP and MCAv, and a greater phase indicates more efficient CA whereby MCAv and BP waveforms are in sync with one another [49]. In the context of our findings, this implies that despite a greater magnitude between MCAv and BP, MCAv response time is improved to maintain synchronised wave forms and ensure intact CA during pregnancy. In the non-pregnant state, the normal boundaries of CA lie between pressures of ~ 60–150 mmHg [50]. Gestational animal models have shown that pregnancy results in an extension of the upper and lower limits of CA [8] and likely occurs to prepare and protect the maternal brain against possible acute and drastic fluctuations in blood pressure [50]. Our data broadly support the animal data in that CA is altered during healthy pregnancy and a larger study would provide sufficient estimates of the upper and lower ranges in healthy pregnancy.

Like other studies, we have shown resting MCAv declines [7]. The reason for MCAv reduction during pregnancy has been attributed to reduced systolic blood pressure [51] and downstream vasodilation of resistance vessels to help maintain a stable hemodynamic state [7, 52]. Although systolic blood pressure was similar during each trimester, carotid artery diameter increased during pregnancy. As an extracranial vessel, an increase in carotid artery diameter reflects reduced vascular resistance and thus implicates blood flow velocity providing further insight and support for pregnancy-related reductions in MCAv. Although not measured in this study, it is plausible that MCA diameter may have increased further to carotid artery enlargement. This may further explain decreased MCAv, although this remains speculative (Tables 5, 6 and 7).

**Table 5:** Maternal cerebral blood flow, neurovascular coupling and cerebrovascular reactivity data for participants in the exercise and control group at the end of trimester 1, 2 and 3

	Exercise group (*N* = 7)			Control group (*N* = 11)			Two-way ANOVA		
Trimester	T1	T2	T3	T1	T2	T3	Time	Intervention	Time × intervention
PCAv (cm s^−1^)	43.7 ± 9.1	45.5 ± 10.6	42.0 ± 5.3	43.5 ± 9.3	38.3 ± 4.9	35.8 ± 4.8	0.15	*0.04*	0.44
NVC response (%)	13 ± 6	14 ± 8	15 ± 9	14 ± 9	17 ± 8	18 ± 4	0.48	0.37	0.85
MCA CVC (cm s^−1^ mmHg^−1^)	0.92 ± 0.14	0.93 ± 0.28	0.87 ± 0.28	0.84 ± 0.13	0.87 ± 0.13	0.74 ± 0.11	0.46	0.07	0.84
MAP (mmHg)	84 ± 10	79 ± 18	83 ± 17	89 ± 11	84 ± 8	90 ± 12	0.42	0.16	0.96
PET CO_2_ (mmHg)	31.9 ± 1.8	33.8 ± 1.9	32.8 ± 1.5	33.0 ± 1.9	32.7 ± 2.1	32.8 ± 0.9	0.82	0.58	0.56
CO_2_ reactivity test									
Carotid diameter (mm)	0.64 ± 0.01	0.66 ± 0.02	0.66 ± 0.01	0.64 ± 0.01	0.66 ± 0.02	0.67 ± 0.01	0.19	0.89	0.72
Carotid diameter (mm) (last 30 s)	0.66 ± 0.02	0.67 ± 0.02	0.67 ± 0.02	0.66 ± 0.01	0.67 ± 0.02	0.68 ± 0.01	0.51	0.74	0.96
Absolute CVR (cm s^−1^ mmHg^−1^)	4.7 ± 1.4	4.4 ± 1.1	4.7 ± 1.5	4.3 ± 1.2	4.1 ± 1.0	3.6 ± 1.1	0.71	0.11	0.58
Relative CVR (cm s^−1^ mmHg^−1^)	4.9 ± 1.4	4.9 ± 1.4	4.7 ± 1.4	4.3 ± 1.2	3.9 ± 0.9	4.3 ± 1.4	0.92	0.23	0.47
CVR (*r*^2^)	0.9 ± 0.1	0.9 ± 0.1	0.8 ± 0.2	0.9 ± 0.1	0.9 ± 0.1	0.8 ± 0.1	0.23	0.85	0.98

Values are mean ± SD. Italics represent significant main effect (*P* < 0.05)

*T1* trimester 1, *T2* trimester 2, *T3* trimester 3, *PCAv* posterior cerebral artery velocity, *NVC* neurovascular coupling, *MCA* middle cerebral artery, *CVC* cerebrovascular conductance, *MAP* mean arterial pressure, *PETCO*_*2*_ end-tidal carbon dioxide, *CVR* cerebrovascular reactivity

**Table 6:** Maternal cerebral autoregulation data for participants in the exercise and control group at the end of trimester 1, 2 and 3

	Exercise group (*N* = 7)			Control group (*N* = 11)			Two-way ANOVA		
Trimester	T1	T2	T3	T1	T2	T3	Time	Intervention	Time × intervention
MAP	99 ± 11	98 ± 10	98 ± 9	101 ± 8	98 ± 10	98 ± 9	0.67	0.81	0.96
PETCO_2_	32.0 ± 2.8	34.7 ± 1.5	34.6 ± 1.4	33.8 ± 2.0	35.0 ± 1.1	35.8 ± 0.8	0.85	0.68	0.66
Gain (cm s^−1^ mmHg^−1^)	1.2 ± 0.3	1.3 ± 0.3	1.0 ± 0.1	0.9 ± 0.3	1.1 ± 0.3	1.0 ± 0.3	0.13	*0.04*	0.34
Phase (degrees)	21.1 ± 10.6	39.6 ± 8.4	34.7 ± 9.1	32.9 ± 13.2	32.8 ± 12.9	47.1 ± 29.4	0.06	0.26	0.10
Normalised gain (%cm s^−1^% mmHg^−1^mmHg/%)	1.6 ± 0.5	1.9 ± 0.5	1.6 ± 0.3	1.3 ± 0.4	1.8 ± 0.5	1.6 ± 0.4	0.08	0.22	0.71
Coherence	0.66 ± 0.07	0.58 ± 0.16	0.62 ± 0.10	0.54 ± 0.14	0.63 ± 0.09	0.61 ± 0.09	-	-	-

Values are mean ± SD. Italics represent significant main effect (*P* < 0.05)

*T1* trimester 1, *T2* trimester 2, *T3* trimester 3

**Table 7:** Offspring characteristics at birth and carotid artery measurements post-partum

	Exercise group (*N* = 7)	Control group (*N* = 11)	*p* value
Male gender	3	6	-
Gestational age at birth (weeks)	39.7 ± 1.1	39.3 ± 1.4	0.72
Weight (kg)	3.5 ± 0.5	3.5 ± 0.4	0.50
Week of IMT measurement PP	8 ± 3	6 ± 2	0.24
IMT (mm)	0.45 ± 0.06	0.49 ± 0.07	0.27
Lumen (mm)	2.92 ± 0.13	2.88 ± 0.40	0.81
IMT/lumen	0.15 ± 0.02	0.17 ± 0.04	0.19

Values are mean ± SD

The brachial and femoral arteries exhibited heterogeneous responses to pregnancy. In line with previous findings, brachial artery diameter increased [53] and femoral artery diameter was unchanged. To date no previous study has examined femoral artery adaptation to a healthy uncomplicated pregnancy. We did observe a decline in the function of the femoral but not the brachial artery. This may be explained by the effect of increased body mass on the vascular beds. Previous authors have reported the femoral artery to be strongly correlated with BMI in comparison to the brachial artery [54]. While the new exercise guidelines for pregnant women may be more effective in improving vascular function in line with previous research [16], our intervention was effective in increasing brachial but not femoral artery blood flow in the exercise group. This adaptation may be indicative of a conditioning effect on the vasculature resulting from repeated exercise bouts that influence shear stress on the vascular wall [55]. Nonetheless, this finding highlights the complexity and heterogeneity of the vascular tree and we suggest simultaneous cerebrovascular and peripheral vascular assessments be used in future randomised controlled trials.

We were also interested whether structured exercise impacts cerebro and peripheral vascular responses during pregnancy. This was of interest due to the benefit of exercise on reducing the incidence of pregnancy-related CV complications including gestational diabetes and pre-eclampsia [56]. The exercise group demonstrated positive changes in some cerebrovascular markers, including autoregulation and neurovascular coupling. Our directional data suggest the exercise group preserved fitness to a greater extent compared with the CONT group.

A novel aspect of this study was the investigation of offspring artery wall thickness within 12 weeks of delivery. We have demonstrated that performing vascular structural assessment at the carotid artery in human offspring is feasible. We show a lower directional offspring IMT following maternal exercise and provide estimates of sample size for a fully powered randomised control trial. Previous animal models have shown a programming effect in vascular function in offspring of exercising mothers [18, 57]. Porcine maternal aerobic exercise training, comprising treadmill running 5 days a week at 60–85% MHR, improves offspring thoracic aorta endothelial cell function measured in vitro at 48 h after birth [18]. The authors used a high exercise dose and it is possible that the new exercise guidelines for pregnant women may provide a greater stimulus for in utero vascular adaptation. Elsewhere, intrauterine growth restriction results in thicker abdominal aortic IMT compared with controls implying the neonatal environment to influence the development of the vascular system [58] further eluding to vascular programming in utero. Taken together, further research in human pregnancy is warranted.

This pilot study has a number of noteworthy strengths including the employment of recommended methodology guidelines for assessment of CA where the dynamic CA regulatory system can be maximally activated [28], FMD [59] and previously published exercise guidelines in pregnancy [20]. This study has achieved the aim of obtaining estimates for the change in maternal cerebrovascular (primary) and offspring vascular function (secondary) during healthy pregnancy that includes structured exercise. This will likely benefit future randomised controlled trials within this population and help advance current knowledge regarding pregnancy-related systemic vascular health. From the pilot study, we have also gain insight for future randomised control trials. Our recruitment rate of 0.7 participants per month from a single site and intervention was via patient choice. We suggest that future trials should aim to maximise recruitment through the use of multi-centre recruitment sites and establishing clinical connections.

## Study Limitations

Firstly, this trial is limited by the recruitment of women at the end of their first trimester of pregnancy meaning we were unable to obtain a pre-pregnancy baseline. Although logistically extremely challenging, future interventions may look to recruit women prior to pregnancy in order to truly understand the impact of pregnancy of systemic vascular function and structure. We failed to observe any differences between groups for PA that may be due to the following limitations/factors: the exercise guidelines employed within this study have been updated in the UK since the commencement of this trial [60] with current guidelines recommending 150 min of moderate intensity PA each week. It is plausible that the exercise stimulus used in this study was too low to influence the CV and lifestyle outcomes assessed between the exercise and control group. Furthermore, it is plausible that not all PA including stationary cycling and water-based activities have been captured by our objective assessment. Future studies would benefit from using an exercise training diary in addition to objective measures. Thirdly, our study is predisposed to recruitment bias as it was likely to appeal to women interested in health and exercise which may explain the similar PA levels observed between groups. The aerobic capacity test used in this study has not been validated in pregnancy and may have overestimated VO_2peak_ due to pregnancy-related increases in heart rate.

In summary, our preliminary evidence suggests that MCAv declines during pregnancy due to reduced vascular resistance and possibly, altered CA. Exercise during pregnancy has some positive effect on maternal and offspring vascular health that should be examined in a larger randomised control trial utilising the estimates from this study to calculate sample size required.

## Data Availability

The authors support data transparency.
